# Magnetic Instabilities in the Quasi-One-Dimensional K_2_Cr_3_As_3_ Material with Twisted Triangular Tubes

**DOI:** 10.3390/ma15062292

**Published:** 2022-03-20

**Authors:** Armando Galluzzi, Giuseppe Cuono, Alfonso Romano, Jianlin Luo, Carmine Autieri, Canio Noce, Massimiliano Polichetti

**Affiliations:** 1 Department of Physics “E.R. Caianiello”, University of Salerno, Via Giovanni Paolo II 132, Fisciano, I-84084 Salerno, Italy; alromano@unisa.it (A.R.); cnoce@unisa.it (C.N.); 2 CNR-SPIN Salerno, Via Giovanni Paolo II 132, Fisciano, I-84084 Salerno, Italy; autieri@magtop.ifpan.edu.pl; 3 International Research Centre Magtop, Institute of Physics, Polish Academy of Sciences, Aleja Lotników 32/46, PL-02668 Warsaw, Poland; gcuono@magtop.ifpan.edu.pl; 4 Beijing National Laboratory for Condensed Matter Physics and Institute of Physics, Chinese Academy of Sciences, Beijing 100190, China; lluo@iphy.ac.cn; 5 Songshan Lake Materials Laboratory, Dongguan 523808, China; 6 School of Physical Sciences, University of Chinese Academy of Sciences, Beijing 100190, China

**Keywords:** Cr-based material, K_2_Cr_3_As_3_, DC magnetic characterization, magnetic hysteresis loops, magnetic instabilities, magnetic frustration

## Abstract

The magnetic response of a frustrated K_2_Cr_3_As_3_ sample having triangular arrays of twisted tubes has been studied by means of dc magnetization measurements as a function of the magnetic field (*H*) at different temperatures ranging from 5 K up to 300 K. Looking at the magnetic hysteresis loops *m*(*H*), a diamagnetic behavior of the sample was inferred at temperatures higher than 60 K, whereas at lower temperatures the sample showed a hysteresis loop compatible with the presence of ferrimagnetism. Moreover, spike-like magnetization jumps, both positive and negative, were observed in a narrow range of the magnetic field around 800 Oe, regardless of the temperature considered and they were compared with the theoretical predictions on frustrated systems. The field position of the magnetization jumps was studied at different temperatures, and their distribution can be described by a Lorentzian curve. The analogies between the expected features and the experimental observations suggest that the jumps could be attributed to the magnetic frustration arising from the twisted triangular tubes present in the crystal lattice of this compound.

## 1. Introduction

In the last few years, the quasi-one-dimensional chromium-based superconductor K_2_Cr_3_As_3_ [1] has been extensively studied. Indeed, it exhibits many interesting properties, regarding the interplay between magnetism, structural properties, superconductivity and quasi-one-dimensionality, shared with other compounds of the family A_2_Cr_3_As_3_ (where A=Na [2], Rb [3], and Cs [4], as opposed to K) and with related compounds belonging to the series ACr_3_As_3_ [5,6]. For completeness, we mention that compounds having the same crystal structure but based on molybdenum A_2_Mo_3_As_3_ [7,8,9,10] have been discovered and other Cr-based superconductors, such as the CrAs, have recently been intensively studied [11,12,13,14,15,16]. Regarding the magnetic properties of K_2_Cr_3_As_3_ and similar materials, both theoretical [17,18] and experimental [1] studies suggest that the ground state is non-magnetic. Theoretically, the nonmagnetic ground state of K_2_Cr_3_As_3_ was attributed to the triangular configuration of the chains composed of chromium atoms which leads to frustration. Experimentally, it has been found that these systems are paramagnetic with small hysteresis loops at low temperature (*T* ≈ 10 K), attributed to defects or magnetic impurities [4], probably due to the presence of KCr_3_As_3_, which instead is magnetic and presents a cluster spin-glass state [5]. The magnetic frustration arises from the presence of twisted triangular tubes with Cr atoms at the vertex of the triangle. Nevertheless, there are theoretical speculations that show that K_2_Cr_3_As_3_ possesses strong magnetic fluctuations and is close to a non-collinear magnetic ground state in–out [19]. Additionally, nuclear quadrupole resonance indicates that moving along the series A = Na, Na_0.75_K_0.25_, K, Rb, the system tends to approach a possible ferromagnetic quantum critical point [20]. However, neutron scattering measurements have established the presence of phonon instabilities related to structural distortions in K_2_Cr_3_As_3_ [21]. These distortions from the triangular structure make the system no longer frustrated. Very recently, we have shown that there is a strong interplay between the structural distortions and magnetism, and we have found that, for the distorted structure, a collinear intrachain ferrimagnetic ground state for the KCr_3_As_3_ and an instability towards the same collinear intrachain ferrimagnetic phase for K_2_Cr_3_As_3_ [22,23]. Assuming intrachain ferrimagnetism, the system could exhibit long-range ferrimagnetism or spin-glass phase depending on the interchain magnetic coupling. Our results predict that K_2_Cr_3_As_3_ is nonmagnetic but close to a long-range ferrimagnetic phase, while the KCr_3_As_3_ goes in a spin-glass phase. Interestingly, when the K_2_Cr_3_As_3_ is considered, the application of a strain can drive the compound from one phase to another, since the non-magnetic phase and the ferrimagnetic one are close in energy [23].

Here, we study a Cr-based quasi-one-dimensional sample containing K_2_Cr_3_As_3_ by means of dc magnetization measurements as a function of magnetic field *H* at different temperatures, ranging from 5 K up to 300 K. Looking at the magnetic hysteresis, we find that at low temperatures the sample shows a ferrimagnetic behavior while at temperatures higher than 60 K a diamagnetic behavior is induced. Since we know that magnetization jumps are theoretically predicted in frustrated quantum spin lattices when the magnetic field is ramped, we have directed our attention to verifying the presence of such magnetization jumps in our sample. In this framework, several spike-like magnetization jumps have been found in a range of −1000 Oe < *H* < 1000 Oe, and this can be explained as due to magnetic instabilities. This paper is organized as follows: in the next section, the experimental results are presented, Section 3 is devoted to the results and discussion whereas the last one contains the conclusions.

## 2. Materials and Methods

A needle-shaped K_2_Cr_3_As_3_ single crystal with a length and thickness equal to 2.5 mm and 0.1 mm, respectively, was analyzed. The fabrication details are reported elsewhere [1,24]. The composition of our sample is K_1.81_Cr_3.57_As_3_. Therefore, the ratio between potassium and chromium is 0.507, which is almost halfway between 0.333 (ratio of the KCr_3_As_3_) and 0.666 (ratio of the K_2_Cr_3_As_3_). So, we can assume that the sample is composed of sizeable parts of both KCr_3_As_3_ and K_2_Cr_3_As_3_, as already reported in the literature [25], which are both superconducting with a *T_c_* = 5 K and *T_c_* = 6.1 K, respectively [1,6].

The sample was characterized in a dc magnetic field applied perpendicularly to its length. In particular, the dc magnetic moment as a function of the field *m*(*H*) was measured using a Quantum Design PPMS-9T equipped with a VSM option. To avoid the effect on the sample response due to the residual trapped field inside the PPMS dc magnet [26], this field was reduced to below 1·10^−4^ T [27]. For what concerns the *m*(*H*) measurements, the sample was first cooled down to the measurement temperature (5 K, 15 K, 30 K, 55 K, 60 K, 100 K, and 300 K) in zero field and thermally stabilized for at least 20 min [28,29]. Then, the field was ramped up to +9 T, then back to −9 T, and finally to +9 T again to acquire the complete hysteresis loop [30,31].

## 3. Results and Discussion

**Figure 1:**
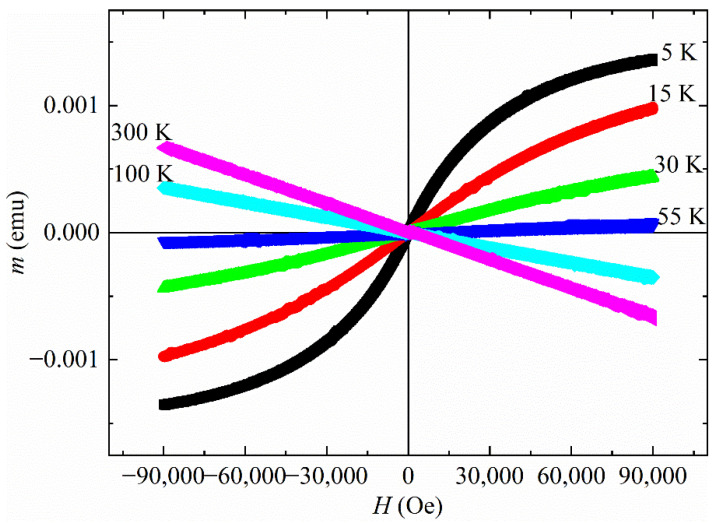
*m*(*H*) curves at different temperatures.

In order to study the magnetic response of the sample, the magnetic moment *m* was measured as a function of the magnetic field *H* in a temperature range from 5 K up to 300 K. In Figure 1, some of the measured *m*(*H*) curves are reported.

**Figure 2:**
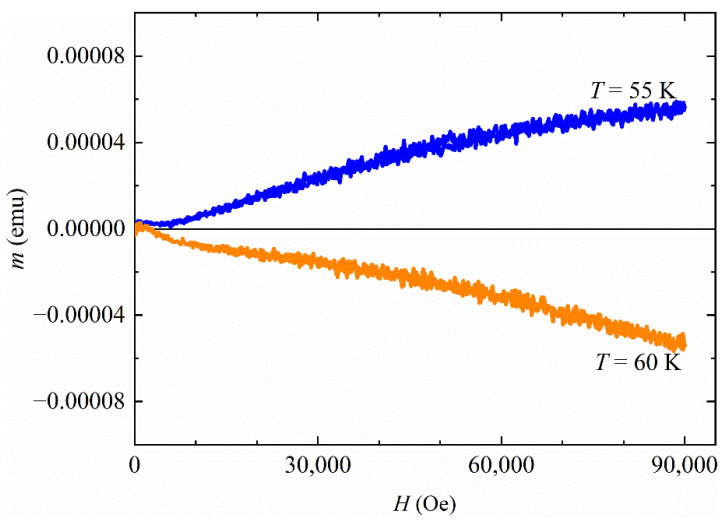
*m*(*H*) curves at *T* = 55 K and *T* = 60 K.

It can be noted that up to 55 K the *m*(*H*) curves show a magnetic behavior that could be associated with the paramagnetism, ferromagnetism or ferrimagnetism phenomenology. The presence of a superconducting phase is not visible because the measurement at the lowest temperature (*T* = 5 K) is very close to the nominal *T_c_* of both phases and the applied field can be high enough to suppress the superconducting state. For *T* = 60 K, the sample is in a diamagnetic state as reported in Figure 2. This suggests a magnetic transition temperature at the 55 K < T* < 60 K.

**Figure 3:**
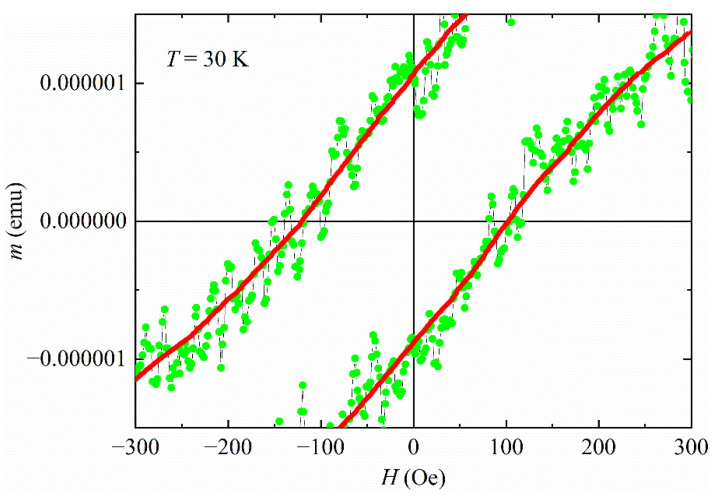
The region near-zero field has been magnified for the *m*(*H*) curve at *T* = 30 K. The red solid line is a guide for the eye.

**Figure 4:**
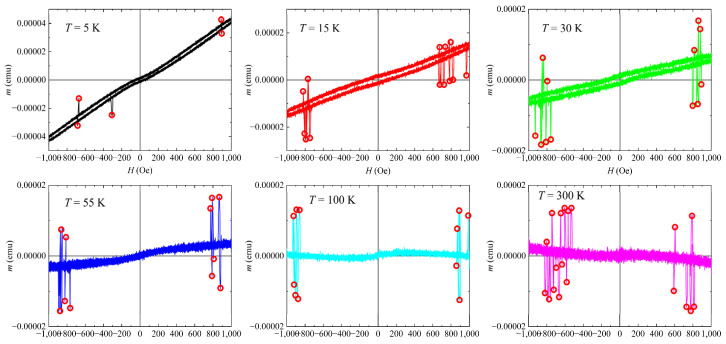
*m*(*H*) curves in the range −1000 Oe < *H* < +1000 Oe. Several spike-like magnetization jumps (indicated by open red circles) can be observed at different fields for all the reported temperatures.

In Figure 3, the *m*(*H*) at *T* = 30 K is reported, focusing on the region near-zero field for evaluating the possible existence of coercivity. It is well visible how the coercive field is different from zero (H_c_ ≈ 100 Oe), highlighting that there is no possibility of a paramagnetic behavior. It is worth noting that H_c_ ≠ 0 Oe was obtained for all the temperatures below 60 K (see also Figure 4). Since for *T* ≥ 60 K the sample is diamagnetic, a coexistence of two sublattices characterized by two opposite magnetic orderings is plausible. This situation is not in contrast with a ferrimagnetic behavior, in agreement with the results reported in Refs. [22,23]. We point out that our measurements are bulk ones, so the results obtained are not due to surface effects.

An interesting feature of the *m*(*H*) curves reported in Figure 1 is visible by focusing on the magnetic response of the sample in the field range −1000 Oe < *H* < +1000 Oe. In fact, several spike-like magnetization jumps, both positive and negative, were observed in different fields, regardless of the temperature considered (see Figure 4). The red circles drawn in the panels of Figure 4 individuate the field position of the magnetization jumps. It is important to highlight that the magnetization jumps are visible for all the reported temperatures independently of the magnetic state that characterizes the sample and independently of the positive or negative values of the applied magnetic field.

**Figure 5:**
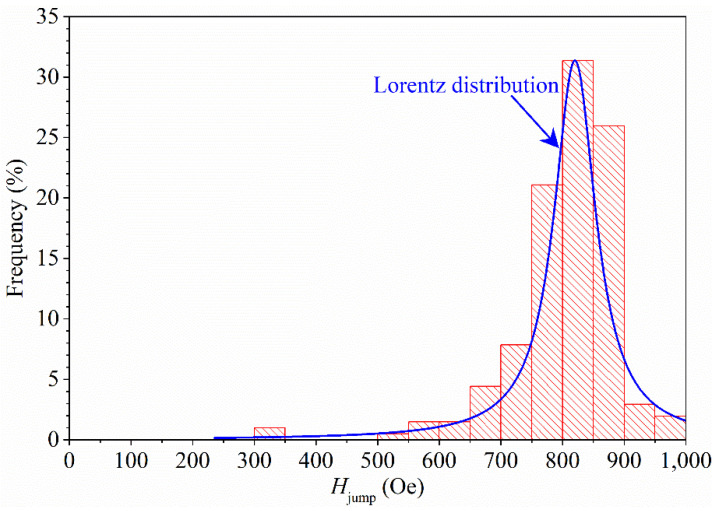
Distribution of the *H*_jump_ values. The blue curves represent the fit of the data with a Lorentz distribution curve. The event at approximately *H*_jump_ = 320 Oe is an outlier of the distribution and is present only in the measurement at lowest temperature.

Such jumps can arise for different reasons. One possibility is a first-order transition between different ground states such as the spin flop transition in classical magnets or in strongly anisotropic quantum chains [32]. Another possibility is related to a macroscopically large degeneracy in the exact ground states of the full quantum system, for some values of the applied magnetic field [33,34,35]. This last option is a quite general phenomenon emerging in highly frustrated systems. Interestingly, it has theoretically been reported that the jumps may occur just below saturation and should be observable in magnetization experiments if the coupling constants are small enough to make the saturating field accessible [33]. Therefore, as the lattice structure of the material here investigated contains twisted tubes triangularly arranged [22,23], we are confident that the jumps observed in our samples originate from the geometrical frustration of the crystal structure of K_2_Cr_3_As_3_. However, in our compound, we have an additional dynamical effect that makes the magnetic state after the jump energetically unstable. As a result, we have spikes in magnetization. These jumps in magnetization were calculated to be of the order of 0.003 Bohr magneton per Cr atom; therefore, they are relatively small compared to the magnetic moments that the Cr atoms can reach. Theoretical calculations [22] predict K_2_Cr_3_As_3_ to be nonmagnetic but on the verge of magnetism, sustaining interchain ferromagnetic spin fluctuations while the intrachain spin fluctuations are antiferromagnetic. In this regime, the magnetic moment of the Cr ion is strongly suppressed compared to the bare Cr magnetic moment. In our material, the Cr atom is expected to have a magnetic dipole moment equal to 3.3 Bohr magnetons [23], so the found value of 0.003 Bohr magneton suggests that only a fraction of about one-thousandth of all the Cr atoms participate in the magnetization jump. It is also worth pointing out that the presence of the same magnetization jumps was verified on other samples of the same batch obtaining the same results, and several tests were performed in order to exclude the presence of artifacts due to the experimental apparatus (see Supporting Material file). The field positions of the magnetization jumps (*H*_jump_) were extracted from all the panels of Figure 4 and a histogram was constructed by setting the frequency expressed as a percentage (%) on the *y* axis and the *H*_jump_ values (taken as the absolute value) on the *x* axis. The result is reported in Figure 5. Each bin has a width of 50 Oe with the highest one ranging between 800 Oe and 850 Oe and showing a 30% presence with respect to the total values.

It is important to note that the interval ranging between 700 Oe and 900 Oe contains more than the 85% of the total values, indicating a narrow field region where most of the magnetization jumps occur. This suggests the presence of magnetic processes activating in correspondence with a specific magnetic energy, allowing the magnetization to jump from a magnetic state to another one. The magnetic energy associated with the measured magnetization jump was evaluated starting from the hypothesis that, as mentioned before, just approximately one-thousandth of all the Cr atoms present in the sample participate in the phenomenon. As a result, we obtained magnetic energy values at *H*_jump_ which are comparable with the thermal energy ones calculated in the temperature range considered for our measurements. The distribution reported in Figure 5 can be well fitted by a Lorentzian curve (peak average ≈820 Oe), which usually characterizes the behavior observed in the absorption spectra.

## 4. Conclusions

To study the magnetic response of a K_2_Cr_3_As_3_ sample, dc magnetization measurements were performed as a function of the magnetic field at different temperatures ranging from 5 K up to 300 K. By analyzing the magnetic hysteresis loops *m*(*H*), we found a magnetic transition at *T* ≈ 60 K from a diamagnetic state (*T* ≥ 60 K) to a paramagnetic response (*T* < 60 K) compatible with the overall ferrimagnetic behavior of the sample. Moreover, focusing on the magnetic response of the sample in the field range −1000 Oe < *H* < +1000 Oe, we found magnetization jumps in a narrow range of magnetic fields. These jumps, theoretically predicted in step-like form, in our case were observed experimentally in the form of spikes, and this behavior is compatible with the presence of magnetic frustrations in quantum spin lattices. The field positions of the magnetization jumps were extracted at different temperatures, reported in a histogram, and then fitted by a Lorentzian distribution curve. We found that more than 85% of the values were included in the field range 700 Oe ≤ *H* ≤ 900 Oe, suggesting that the lattice needs a specific magnetic energy to allow the magnetization to jump from a magnetic state to another one. Further investigations on the dynamical effect that makes the observed jumps unstable are necessary to fully address these new findings.

## Data Availability

The data sets that support the findings in this study are available from the corresponding author upon reasonable request.

## Supplementary Materials

The following supporting information can be downloaded at: https://www.mdpi.com/article/10.3390/ma15062292/s1, Figure S1: Spike-like magnetization jumps; Figure S2: Artifacts due to experimental apparatus.
