# Supplementary material for: Magnetic Instabilities in the Quasi-One-Dimensional K2Cr3As3 Material with Twisted Triangular Tubes
Source: Materials (Basel). 2022 Mar 20;15(6):2292. doi: 10.3390/ma15062292 (PMC8954554; doi:10.3390/ma15062292)
Supplement: Supplementary file 1 [file materials-15-02292-s001.zip › materials-1621486-supplementary.pdf]

[Supplementary Materials] Magnetic instabilities in the quasi-one-dimensional $K_2Cr_3As_3$ material with twisted triangular tubes

Armando Galluzzi[1,2,*], Giuseppe Cuono[3], Alfonso Romano[1,2], Jianlin Luo[4,5,6], Carmine Autieri[2,3], Canio Noce[1,2] and Massimiliano Polichetti[1,2,*]

[1] Department of Physics "E.R. Caianiello", University of Salerno, via Giovanni Paolo II, 132, Fisciano (SALERNO), I-84084, Italy; agalluzzi@unisa.it (A.G.); alromano@unisa.it (A.R.); cnoce@unisa.it (C.N.); mpolichetti@unisa.it (M.P.)
[2] CNR-SPIN Salerno, via Giovanni Paolo II, 132, Fisciano (SALERNO), I-84084, Italy; autieri@magtop.ifpan.edu.pl (C.A.)
[3] International Research Centre Magtop, Institute of Physics, Polish Academy of Sciences, Aleja Lotników 32/46, PL-02668 Warsaw, Poland; gcuono@magtop.ifpan.edu.pl (G.C.);
[4] Beijing National Laboratory for Condensed Matter Physics and Institute of Physics, Chinese Academy of Sciences, Beijing 100190, China; lluo@iphy.ac.cn (J.L.)
[5] Songshan Lake Materials Laboratory, Dongguan, Guangdong 523808, China
[6] School of Physical Sciences, University of Chinese Academy of Sciences, Beijing 100190, China
* Correspondence: agalluzzi@unisa.it (A.G.); mpolichetti@unisa.it (M.P.)

Spike-like magnetization jumps

The m(H) measurement has been performed at T = 300 K on a second $K_2Cr_3As_3$ sample from the same batch in order to verify the presence of the same magnetization jumps. In Figure 1, the results for the sample of the Manuscript are shown in the upper panel while the results obtained for the second sample from the same batch are reported in the lower panel. It is clearly visible how the second sample results are very similar to the manuscript sample ones showing the presence of the same magnetization jumps in the same field region.

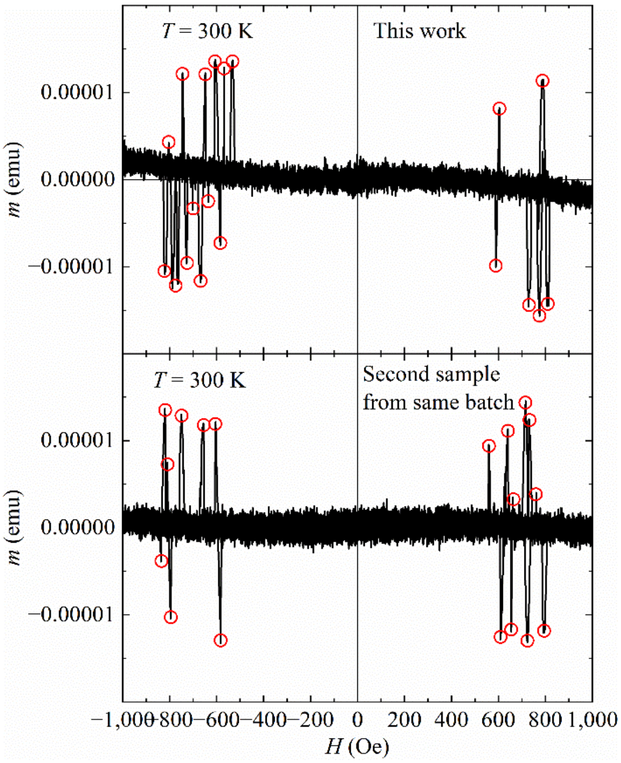

Figure S1. m(H) curves in the range -1000 Oe < H < +1000 Oe for the $K_2Cr_3As_3$ of the Manuscript (upper panel) and for a second $K_2Cr_3As_3$ sample from the same batch (lower panel). The presence of the magnetization jumps is visible for both the samples.

Artifacts due to experimental apparatus

As reported in the main text of the Manuscript, several tests were carried out in order to exclude the presence of artifacts due to the experimental apparatus. In particular, together with the second $K_2Cr_3As_3$ sample taken from the same batch, other two samples have been measured (CuNi and PdNi) focusing our attention on the magnetic field region where the magnetization jumps appear, i.e., -1000 Oe and +1000 Oe. The results are reported in Figure 2 where the three m(H) curves have been plotted together to better show their features. It is evident that the magnetization jumps appear only for the $K_2Cr_3As_3$ sample while both CuNi and PdNi do not show any magnetization jump in the same field region. This points out that the magnetization jumps are a feature of the sample studied in the Manuscript and they are not due to the presence of artifacts originating from the experimental apparatus.

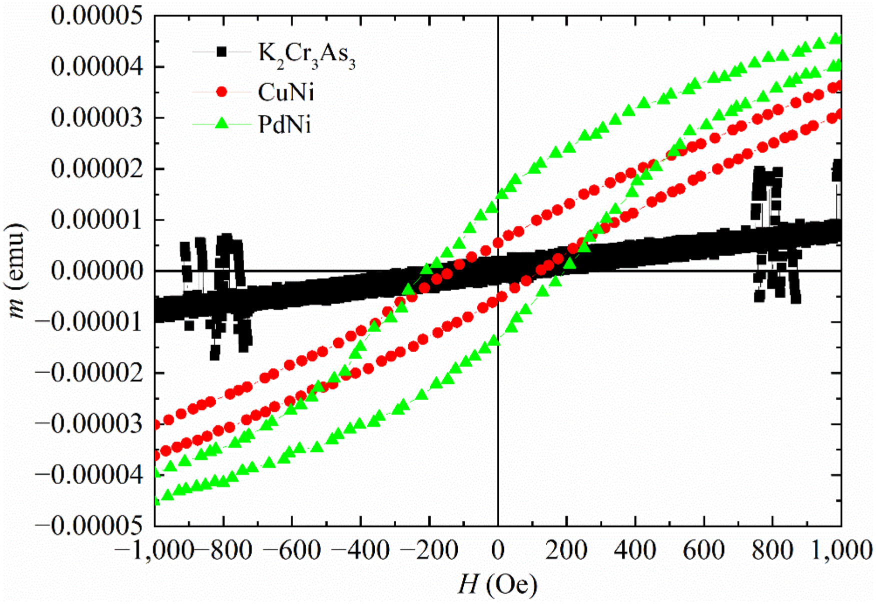

Figure S2. m(H) curves in the range -1000 Oe < H < +1000 Oe for $K_2Cr_3As_3$ (black squares), CuNi (red circles), PdNi (green triangles). The presence of the magnetization jumps is visible only for the $K_2Cr_3As_3$.
